# Relative Expression of Peptidylarginine Deiminase 2 and Sex Steroid Receptors in XX and XY Mouse Placenta

**DOI:** 10.3390/ijms262110523

**Published:** 2025-10-29

**Authors:** Amanda Wewer, Autumn Bennitt, Emily Hinners, Morgan Helmich, Nathan Schnepp, Sean Pitcher, Agata M. Parsons, Gerrit J. Bouma

**Affiliations:** 1 Western Michigan University, Homer Stryker MD School of Medicine, Kalamazoo, MI 49007, USA; amanda.wewer@wmed.edu (A.W.); autumn.bennitt@wmed.edu (A.B.); emily.hinners@wmed.edu (E.H.); morgan.helmich@wmed.edu (M.H.); sean.pitcher@wmed.edu (S.P.); agata.parsonsaubone@wmed.edu (A.M.P.); 2 Department of Biological Sciences, Western Michigan University, Kalamazoo, MI 49007, USA; nathan.p.schnepp@wmich.edu

**Keywords:** placenta, PAD2, peptidylarginine deiminase, androgen receptor, estrogen receptor 1, fetus

## Abstract

Although female (XX) and male (XY) placentas generally function the same, it is evident that there are sex-specific postnatal health outcomes following placental dysfunction and pregnancy complications. Although the underlying causes for these sex differences are unclear, it is postulated that differences in XX and XY placental function are involved due to sex chromosomes and/or sex steroids. Studies in breast and prostate cancer cells demonstrated a role for the citrullination enzyme peptidylarginine deiminase 2 (PAD2) in post-translational regulation of estrogen (ESR) and androgen receptor (AR) signaling. The goal of this study is to determine if PAD2 is present in mouse placentas and if XX versus XY differences exist in the relative level of PAD2. Fetuses and placentas were collected from three pregnant mice (C57BL6) at 14 days of gestation. Total RNA and protein were isolated from XX and XY placentas, and relative mRNA and protein were analyzed by real-time PCR and Western blot. AR and PAD2 levels were significantly higher in XY than in XX placentas. This study is the first to demonstrate XX and XY differences in PAD2 and AR in the placenta. It suggests a role for PAD2 regulation of androgen receptor signaling in the XY placenta.

## 1. Introduction

Pregnancy disorders such as pre-eclampsia, intrauterine growth restriction, and gestational diabetes are accompanied by placental dysfunction, which greatly affects maternal, fetal, and postnatal health [1,2,3]. A temporary and transitory organ, the placenta develops and differentiates rapidly after attachment of the blastocyst to the endometrium and is able to perform its functions at the start of the second trimester. Throughout pregnancy, the placenta secretes hormones that regulate maternal and fetal physiology, are critical for nutrient and gas exchange, and support the growth and development of the fetus.

Although female (XX) and male (XY) placentas generally function the same, there are differences in in utero growth rates between XX and XY fetuses, as well as neonatal adverse outcomes following pregnancy complications. For example, ultrasound scans revealed that the growth rate per day was greater for male fetuses compared to female fetuses [4]. In fact, sex differences in length have already been observed as early as the first trimester [5].

In addition to growth, sexual dimorphic responses have been observed following pregnancy complications associated with placental dysfunction. Stark and colleagues reported significantly greater basal peripheral microvascular blood flow in male compared to female newborns in pregnancies complicated by pre-eclampsia [6]. Similarly, small for gestational age pregnancies associated with stillbirth were significantly higher for males than females [7]. Finally, a retrospective study of pregnancies with normal glucose tolerance and gestational diabetes revealed an increased risk for neonatal infection, acute respiratory disorders, and abnormal central nervous system development in neonatal males from gestational diabetes pregnancies compared to females [8].

It is reasonable to assume that these differences in female and male fetuses are innately due to their sex chromosomes and/or sex steroids, or differences in XX and XY placental function. Sex differences in gene expression in the placenta itself are thought to mediate differences in growth and survival of male and female offspring [9,10,11]. Placental transcriptome profiling data analysis uncovered transcript expression related to increased placental reserve capacity in female placentas, and increased growth in male placentas (reviewed in [11]). Although the exact role for many of these XX/female and XY/male-biased genes in sex-specific differences in placental function is unknown, it is important to recognize that XX and XY placentas are not identical.

In addition to XX and XY genetic differences, differences in sex steroid content within amniotic fluid exist between female (higher estradiol) and male (higher testosterone and delta 4-androstenedione) fetuses [12,13]. Furthermore, testicular differentiation is complete at ~8 weeks of gestation and leads to increased endogenous testosterone production and secretion in XY fetuses. Therefore, female and male differences in fetal growth and development could be attributed to sex steroid signaling in addition to sex chromosomes. Not only is the placenta a source but also a target of sex steroids. Estrogen receptors (ESR1, ESR2) and androgen receptors (AR) are present in placental trophoblast cells (reviewed in [14]), and likely involved in various processes including cell proliferation, differentiation, and angiogenesis [15,16]. Furthermore, in vitro studies indicate different androgen receptor variants are present in human placental tissue and may play a role in regulating fetal growth [17]. Currently, the role of sex steroid receptors in mediating sex differences in placental gene expression and/or function is unknown.

Post-translational modifications regulate gene expression and function by mediating various processes, including transcriptional activation, repression, and chromatin packaging. One form of post-translational modification is citrullination of arginine residues through PAD enzymes. The PAD family of post-translational modification enzymes has five family members, PAD1–PAD4 and PAD6. PAD2 is the ancestral homolog and is widely expressed in mammalian tissues [18,19]. PAD2 converts positively charged arginine into neutral citrulline (called citrullination) in cytoplasmic proteins and on histone tails. Citrullination on histone tails alters chromatin conformation and thereby regulates gene expression. Previous work revealed an abundant presence of PAD2 in prostate and breast cancer as well as uterine tissue [20,21,22]. In prostate cancer cells, PAD2 citrullination of histone 3 regulates AR signaling [21]. In breast cancer cells, PAD2-mediated H3Cit regulates ESR signaling [23].

PAD2 is present in the placenta and has been detected in isolated mouse trophoblast stem cells, where it appears to prevent premature differentiation of trophoblast stem cells into trophoblast giant cells [19,24]. Considering the many similarities between trophoblast and cancer cells, and the presence of PAD2 in the placenta, PAD2 may play an important novel role in the regulation of gene expression and function in placental trophoblast cells.

Our long-term goal is to obtain a better understanding of the regulation of sex steroid receptor signaling in the placenta. In the current study, we aimed to identify the relative expression of AR, ESR1, and PAD2 in mouse placentas and to determine if differences exist between XX and XY mouse placentas. Our data will provide evidence for a novel mechanism regulating sex steroid receptor function in the placenta through citrullination. Ultimately, these data will set the stage for future new studies demonstrating the function of PAD-mediated citrullination in steroid receptor signaling in placental function and pregnancy.

## 2. Results

### 2.1. Pad2, Ar, and Esr1 mRNA in XX and XY Placentas

**Figure 1:**
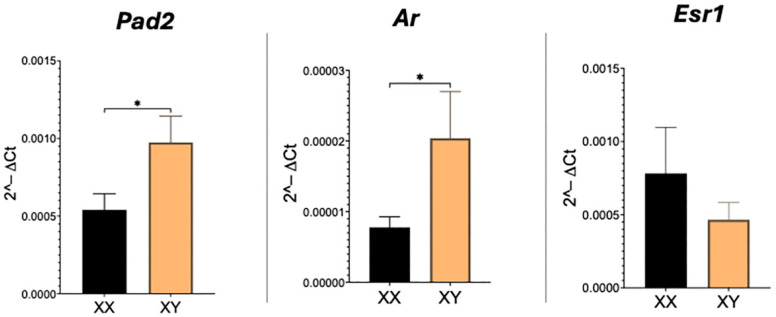
Real-time PCR results for Pad2, Ar, and Esr1 mRNA in XX and XY placentas. Student’s *t*-test: * *p* < 0.05.

As expected, transcripts for Pad2, Ar, and Esr1 are readily detected in the mouse placenta. Real-time PCR revealed that relative amounts of both Pad2 and Ar are significantly (*p* < 0.05) higher and doubled in XY compared to XX placentas (Figure 1). Esr1 transcript levels, however, were not different between XX and XY placentas.

### 2.2. PAD2, AR, and ESR1 Protein in XX and XY Placentas

**Figure 2:**
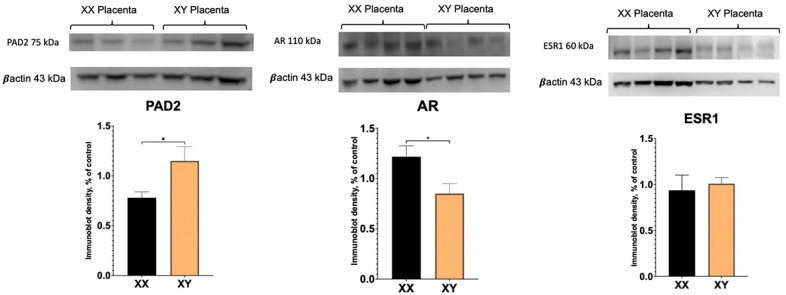
Western blot results for PAD2, AR, and ESR1 protein in XX and XY placentas. Student’s *t*-test: * *p* < 0.05.

To determine if there are sex differences in PAD2 and AR protein between XX and XY placentas, Western blotting was performed. As demonstrated in Figure 2, PAD2 protein amounts were significantly higher in the XY placenta. Unexpectantly, whereas AR transcript level was significantly increased (Figure 1), AR protein amount appeared to be significantly (*p* < 0.05) decreased in XY compared to XX placentas.

## 3. Discussion

It has become increasingly evident that physiological differences exist between XX and XY individuals, including fetal growth. Changes in XX and XY placenta have been reported before; these primarily indicate differences at the genetic and molecular level. Inherently, biological sex differences are due to the presence and contribution of sex chromosomes and/or sex steroid hormones (i.e., estrogens and androgens). In this study, our goal was to explore the potential difference in androgen signaling between the XX and XY placenta. Previous work in prostate cancer cells indicated that PAD2 regulates AR expression and signaling. In this study, our results reveal for the first time a difference in the presence of AR as well as PAD2 between XX and XY in the placenta.

Regulation of PAD2 expression by sex steroids is tissue-specific. Estrogen regulates PAD2 expression in uterine tissue, but sexual dimorphic expression in the aging heart appears independent of estrogen [20,25]. Similarly, PAD2 expression is regulated by androgens in prostate cancer cells, and interestingly, PAD2 regulates the stability and nuclear import of AR protein and AR target gene expression [21]. Considering differences in androgens in amniotic fluid between XX and XY fetuses, it is interesting to speculate that this could drive the sexual dimorphic expression of PAD2.

Alterations in protein citrullination in the placenta correlate with early- and late-stage onset of fetal growth restriction (FGR), indicating that PAD enzymes play a role in placental function [26]. Specifically, late-onset FGR appears to be associated with significantly increased placental PAD2 compared to appropriate for gestational age (AGA) controls. FGR pregnancies are more commonly associated with female fetuses [27,28], suggesting that PAD function could play a sex-specific role in placental function.

Our real-time PCR experiments reveal significantly higher levels of AR transcript in XY compared to XX placentas. However, surprisingly, the AR protein was significantly higher in the XX placenta. In the human and sheep placenta, multiple (seven) AR protein variants have been described using different antibodies [17,29]. In addition to the full-length, ~110 kDa size AR protein, smaller known variants of 76 kDa, 75 kDa, and 45 kDa are also present in human and sheep placental tissue, as well as unknown variants. No XX versus XY sex-specific differences were investigated and/or reported in these studies. Our results reveal a full-length ~110 kDa band that is readily detected in XX and XY mouse placenta and is less abundant in XY compared to XX mouse placentas. The ~75 kDa also is also present in the mouse placenta; however, only a very faint ~45 kDa band could be detected in the mouse placenta (see Supplemental Figure S1). Although the exact function of these multiple protein variants in placental tissue is unclear, in the human placenta, the 45 kDa AR variant was increased, and the 75/76 kDa AR variants were decreased in male-controlled asthmatic pregnancies [17]. Clearly, the presence of multiple slice variants, their presence in different placental cells, and differential regulation by various external factors highlight the complexity of AR signaling in the placenta and warrant further investigation.

In mice, *Ar* reportedly exhibited sexual dimorphic expression with significantly higher transcript levels in the female placenta from mice fed a very high-fat diet compared to the male placenta, according to microarray analysis [30]. Interestingly, this data set also indicates that *Pad2* levels were significantly higher in female placentas. These results indicate maternal environment can have sexual dimorphic effects on placental *Ar* and *Pad2* expression. Moreover, placental *Ar* expression is impacted by fetal size, as lighter male fetuses have significantly elevated placental *Ar* compared to heavier male fetuses [31].

The strengths of this study are that it provides novel data regarding the sexual dimorphic expression of AR and PAD2 in the mouse XX and XY placenta, which provides for future experiments aimed at elucidating the function of AR and PAD2 in the hemochorial placenta. A limitation is that this study is descriptive and highlights the presence of AR and PAD2 at one time point during gestation. Furthermore, the presence and role of additional AR variants and PAD isozymes remains to be determined.

## 4. Materials and Methods

**Tissue Collection:** Four C57BL/6J female mice were used for this experiment. Midday, the day a vaginal plug was present was considered embryo day (E) 0.5. At approximately 13.5 days of gestation, females were euthanized. Individual placentas were dissected, and half of each placenta was snap frozen, and the other half of the placenta was fixed in 4% paraformaldehyde overnight at 4 °C.

**DNA Isolation and Genotyping:** To determine the chromosomal sex of individual fetuses and accompanying placenta, genomic DNA (gDNA) was isolated from fetal tails using the Monarch Genomic DNA Purification Kit (NEB, T3010S, Ipswich, MA, USA) according to the manufacturer’s instructions. Multiplex PCR was performed using primers for the YMT2/B locus on the Y chromosome and primers for the autosomal myogenin gene as a positive control [32]. The PCR amplification cycle was as follows: 95 °C (2 min), followed by 34 cycles at 96 °C (10 s), 60 °C (30 s), 72 °C (30 s), and one cycle at 72 °C (5 min). Following gel electrophoresis, XX tissues were identified by the presence of one amplicon for myogenin, whereas XY tissues were identified by the presence of two amplicons, one for the Y chromosome locus and one for myogenin.

**RNA Isolation:** Total RNA was isolated from 7 XX and 8 XY placentas using the Monarch Total RNA Miniprep Kit (NEB, T2010S, Ipswich, MA, USA) according to the manufacturer’s instructions. RNA was resuspended in 50 μL DNAse-/RNAse-free water. Quantity and purity of RNA were assessed using a NanoDrop One Spectrophotometer, and only samples with 260/280 ratios greater than 1.9 were used. Total RNA was stored at −80 °C until use.

**Real Time PCR:** Total RNA was reverse transcribed into cDNA using the ProtoScript II mix following the manufacturer’s instructions, and 20 μL cDNA was diluted with 50 μL DNA-/RNAse-free water. Real-time PCR reactions were performed in 96-well PCR plates (MicroAmp Optical 96-well reaction plate, Applied Biosystems, Foster City, CA, USA), with each reaction containing 1 μL diluted cDNA, 5 μL PowerTrack SYBR Green Master Mix (PowerUp SYBR Green Master Mix, Applied Biosystems), 1 μL (5.0 μM) forward and reverse gene-specific primers (Table 1), and 3 μL of DNA-/RNAse-free water. Each cDNA sample was loaded in duplicate, and real-time PCR analysis was performed using the QuantStudio 3 Real-Time PCR system. PCR cycle conditions were: 50 °C for 2 min, 95 °C for 2 min, 40 cycles of 95 °C for 15 s and 60 °C for 1 min, and a melt curve of 95 °C for 15 s, 60 °C for 1 min, and 95 °C for 15 s. Experimental replicates were normalized using the geometric mean of actin and 18s rRNA (Pad2 and AR) or Gapdh (Esr1) endogenous reference genes. Experiments were repeated twice.

**Western blot:** Total protein was isolated using the Total Protein Extraction Kit for Animal Cultured Cells and Tissues (Invent Biotechnologies, Inc., Plymouth, MN, USA; SD-001/SN-002) using the manufacturer’s instructions, and protein concentration was determined using a NanoDrop One Spectrophotometer. For Western blotting, 50 μL aliquots of protein sample (containing 30 μg, 2X Laemmli sample buffer (BioRad, Hercules, CA, USA), and 5% β-mercaptoethanol) were boiled for 5 min, and separated on SDS-PAGE (TGX gels, BioRad) run at 130 V for 60 min. Samples were transferred to PVDF membranes, previously soaked in 100% ice-cold methanol, at 40 V for 1 h on ice. Following washes in 1X TBS-T, membranes were blocked in 5% dry milk for 1 h at room temperature. PAD2, AR and ESR1 protein were detected by incubating the membrane in PAD2 polyclonal antibody (12110-1 AP, LS Bio, Seattle, WA, USA), AR monoclonal antibody (sc-7305, Santa Cruz, Dallas, TX, USA), or ESR1 monoclonal antibody (ab32063, Abcam, Cambridge, UK) diluted 1:200, 1:3000, and 1:1000 in TBS-T, respectively, overnight at 4 °C. Membranes were washed in TBS-T (3 × 5 min) and incubated with HRP-conjugated secondary antibody (65-6120, Invitrogen; 31457, ThermoFisher Scientific Inc., Waltham, MA USA, resp.) for 1 h at room temperature. Following washes in TBS-T (3 × 5 min), protein bands were visualized using enhanced chemiluminescence SuperSignal West Dura Extended Duration Substrate kit (37071, ThermoFisher Scientific Inc.) and imaged using a Li-COR (C-Digit) imager. All blots were re-probed with HRP-conjugated anti-β-actin (MAS-15738, Invitrogen, Carlsbad, CA, USA) at 1:4000 dilution for quantification by densitometry using ImageJ version 2.14.0/1.54f. A total of 3–4 different XX and 3–4 different XY placentas were used, and experiments were repeated at least once.

**Statistical Analysis:** Statistical differences in relative mRNA and protein amounts were assessed using GraphPad Prism version 10.5.0. Student’s *t*-test was performed to determine if differences exist between XX and XY placentas. Statistical significance was regarded as *p* < 0.05.

## 5. Conclusions

This study is the first to demonstrate XX and XY differences in PAD2 and AR in the placenta. PAD2 mRNA and protein were significantly higher in XY compared to XX placentas. No significant differences were observed in the abundance of Esr1/ESR1. These data indicate that PAD2 may play a role in AR signaling in XY placentas.

## Figures and Tables

**Table 1:** Primers used in the real-time PCR assay (top) and genotyping assay (bottom).

Primer	Forward (5′ to 3′)	Reverse (5′ to 3′)	Amplicon Size (bp)	Accession Numbers
**Real-Time PCR**				
Pad2	CAGCCGCCTATACGGGAAAA	CCTCCTCTGCCTCTCCATCA	202	ENSMUSG00000028927
ESR1	CCGCAGCTGTCTCCTTTCCT	GTCATTGCACACGGCACAGT	228	ENSMUSG00000019768
AR	GCTGACAAGCCAGGAGAGTGA	TGGGTAAAACATGGTCCCTGGTA	182	ENSMUSG00000046532
Actin	GGCTGTATTCCCCTCCATCG	CCAGTTGGTAACAATGCCATGT	155	M12481.1
18s rRNA	GAGGCCCTGTAATTGGAATGAG	GCAGCAACTTTAATATACGCTATTGG	120	NR_003278.3
Gapdh	AACTTTGGCATTGTGGAAGG	GGATGCAGGGATGATGTTCT	132	NM_001411841
**Genotyping**				
YMT2/B locus	CTGGAGCTCTACAGTGATG	CAGTTACCAATCAACACATCAC	389	XM_017318767.2
Myogenin	TTACGTCCATCGTGGACAGCAT	TGGGCTGGGTGTTAGTCTTAT	269	M95800.1

## Data Availability

All data is available upon reasonable request from the corresponding author.

## Supplementary Materials

The following supporting information can be downloaded at: https://www.mdpi.com/article/10.3390/ijms262110523/s1.

## Abbreviations

The following abbreviations are used in this manuscript:

PAD2	Peptidylarginine deiminase 2
AR	Androgen receptor
ESR1	Estrogen receptor 1
