# Supplementary material for: Relative Expression of Peptidylarginine Deiminase 2 and Sex Steroid Receptors in XX and XY Mouse Placenta
Source: Int J Mol Sci. 2025 Oct 29;26(21):10523. doi: 10.3390/ijms262110523 (PMC12607503; doi:10.3390/ijms262110523)
Supplement: Supplementary file 1 [file ijms-26-10523-s001.zip › ijms-3874262-supplementary.pdf]

International Journal of
Molecular Sciences

Figure S1. Representative Western Blot image of AR protein in placental samples highlighting different splice variants.

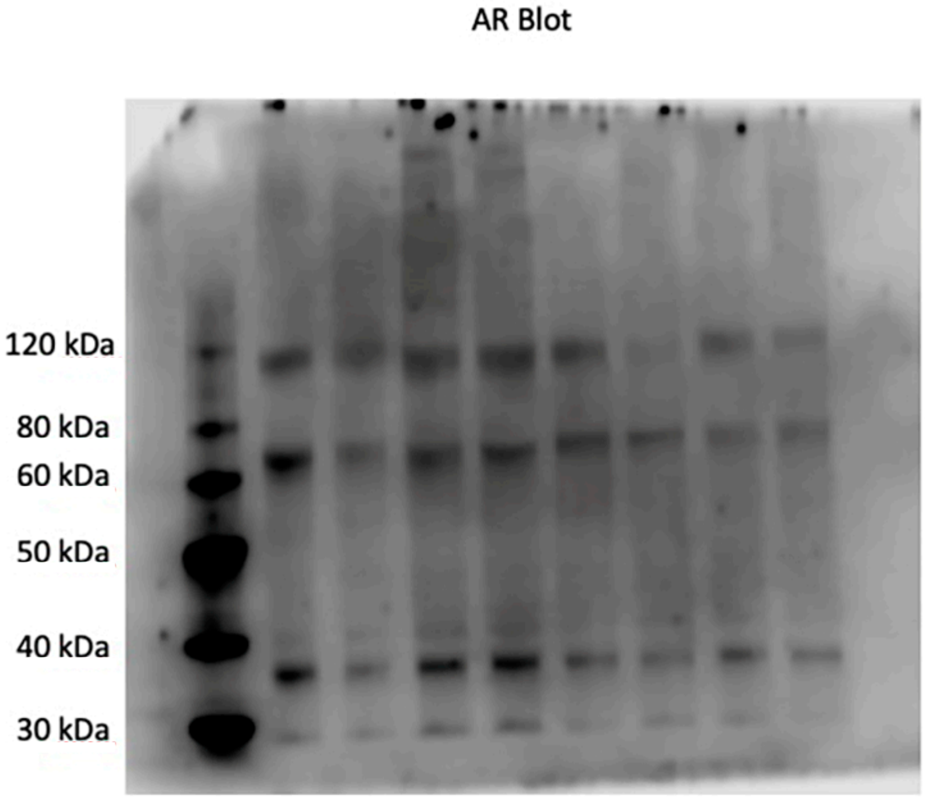

 https://doi.org/10.3390/ijms262110523
